# Therapist-Assisted Rehabilitation of Visual Function and Hemianopia after Brain Injury: Intervention Study on the Effect of the Neuro Vision Technology Rehabilitation Program

**DOI:** 10.2196/resprot.8334

**Published:** 2018-02-27

**Authors:** Rune Skovgaard Rasmussen, Anne Marie Heltoft Schaarup, Karsten Overgaard

**Affiliations:** ^1^ Herlev Hospital Department of Neurology N108 University Hospital of Copenhagen Herlev Denmark; ^2^ The Institute for the Blind and Partially Sighted (IBOS) Hellerup Denmark

**Keywords:** stroke, vision, rehabilitation, brain injury

## Abstract

**Background:**

Serious and often lasting vision impairments affect 30% to 35% of people following stroke. Vision may be considered the most important sense in humans, and even smaller permanent injuries can drastically reduce quality of life. Restoration of visual field impairments occur only to a small extent during the first month after brain damage, and therefore the time window for spontaneous improvements is limited. One month after brain injury causing visual impairment, patients usually will experience chronically impaired vision and the need for compensatory vision rehabilitation is substantial.

**Objective:**

The purpose of this study is to investigate whether rehabilitation with Neuro Vision Technology will result in a significant and lasting improvement in functional capacity in persons with chronic visual impairments after brain injury. Improving eyesight is expected to increase both physical and mental functioning, thus improving the quality of life.

**Methods:**

This is a prospective open label trial in which participants with chronic visual field impairments are examined before and after the intervention. Participants typically suffer from stroke or traumatic brain injury and will be recruited from hospitals and The Institute for the Blind and Partially Sighted. Treatment is based on Neuro Vision Technology, which is a supervised training course, where participants are trained in compensatory techniques using specially designed equipment. Through the Neuro Vision Technology procedure, the vision problems of each individual are carefully investigated, and personal data is used to organize individual training sessions. Cognitive face-to-face assessments and self-assessed questionnaires about both life and vision quality are also applied before and after the training.

**Results:**

Funding was provided in June 2017. Results are expected to be available in 2020. Sample size is calculated to 23 participants. Due to age, difficulty in transport, and the time-consuming intervention, up to 25% dropouts are expected; thus, we aim to include at least 29 participants.

**Conclusions:**

This investigation will evaluate the effects of Neuro Vision Technology therapy on compensatory vision rehabilitation. Additionally, quality of life and cognitive improvements associated to increased quality of life will be explored.

**Trial Registration:**

ClinicalTrials.gov NCT03160131; https://clinicaltrials.gov/ct2/show/NCT03160131 (Archived by WebCite at http://www.webcitation.org/6x3f5HnCv)

## Introduction

Worldwide, stroke is the second most common cause of death and the third most common cause of disability [1]. Serious and often lasting vision impairments affect 30% to 35% of people with stroke [2]. In general, vision may be considered the most important sense in humans, and even small permanent deficits can dramatically affect the quality of life.

Lack of vision (anopia) after brain damage limits rehabilitation and enhances other invalidating effects. Impaired vision results in impaired balance, increased risk of serious falls, increased support needs, reduced quality of life and an impaired ability to perform activities of daily living [3]. Recovery of visual field deficits occurs primarily and only to a modest extent the first month after brain injury [4], and thus the time window for spontaneous improvements is very limited. Hence, brain-impaired persons with visual impairment will most likely experience chronically impaired vision already 4 weeks after brain injury, and the need for visual compensatory rehabilitation is substantial [4].

Neuro Vision Technology is a supervised training course where people with visual field deficits are trained in compensatory and restorative techniques. The course includes a special computer program, a light panel and a special training program, and the course is conducted by occupational therapists with Certified Orientation & Mobility Instruction (O&M instructors) with visual expertise [2]. Through the Neuro Vision Technology course, the individual’s specific vision deficits are carefully identified through a five-step program. Personal data is used to organize individual training and thereby teaching the individual to cope with situations that cause problems in everyday life. In spite of reported positive results of Neuro Vision Technology training among several individuals [5], there are currently no scientific studies [2] on whether rehabilitation with Neuro Vision Technology results in lasting and clinically significant improvements in people with severe and chronic visual field impairments.

The aim and hypothesis of this study is to test whether rehabilitation with Neuro Vision Technology can lead to significant and lasting improvements in persons with chronic visual impairments after brain injury. Improving eyesight is expected to increase both physical and mental functioning, thus improving the quality of life. Study participants will be investigated in terms of both visual and mental functions, including quality of life, cognition and depression.

## Methods

This study is designed as a prospective study in which the same subject is examined before and after the intervention.

### Participants

Participants are suffering from chronic visual field impairments; thus, participants will not experience significant spontaneous remission or deterioration. Participants have typically experienced a stroke (ischemic or hemorrhagic) or traumatic brain injury. There is a high risk of injury worsening in participants with malignant or progressive tumors, making it difficult or impossible to document a beneficial effect of the intervention; Participants with such progressive diseases as well as participants worsening (eg, from secondary strokes and with further visual impairment) will be excluded from participation in the trial.

### Recruitment

Suitable subjects are consecutively included from the Institute for Blind and Partially Sighted, Herlev University Hospital (especially Neurological Department N108), and other neurological departments (see Textbox 1). A screening will be performed to assess the suitability of a person to participate in the trial. Attendance is voluntary and all sessions are free for the participant. Written consent is required.

### Neuro Vision Technology Intervention and Mobility

**Figure 1:**
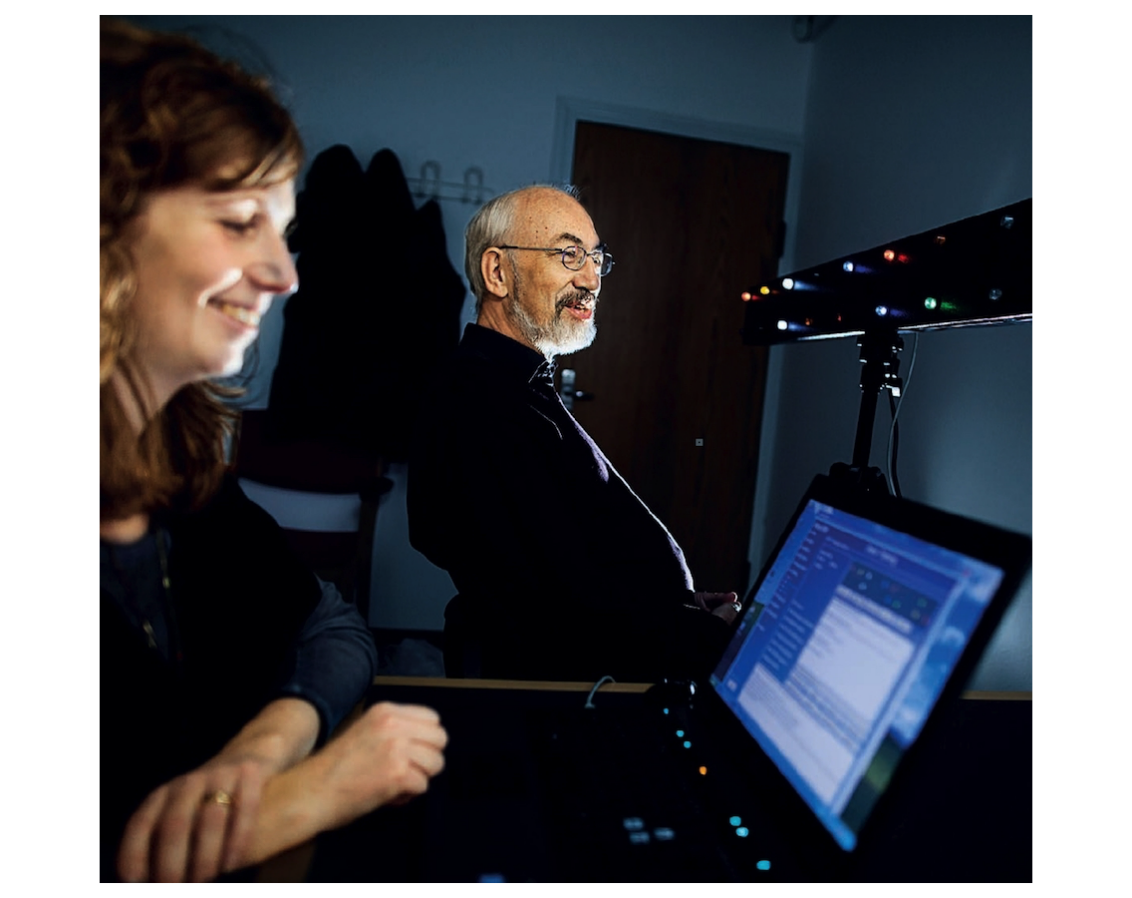
An example of NVT training, where a brain damaged participant in need of vision rehabilitation is given compensatory rehabilitation using a special computer system controlling a light panel.

The term “orientation & mobility” is understood as the ability to move safely in physical space and in the surrounding community. By controlling orientation and mobility techniques, a person with a restricted field of vision can be able to move not only safely, but also freely and independently. The Neuro Vision Technology method uses a special device in the form of light panels and neurological vision tests providing an overview of visual and cognitive difficulties. The light panel is connected to a computer, and an example showing the setup together with a male participant and a female investigator can be seen in Figure 1. Study participants are then placed at the light panel, where the scan pattern is automated and gradually transferred to indoor mobility routes. When the procedures are automated, the exercise is done outdoors using progressively increasing complexity.

A Neuro Vision Technology course includes 3 hours of cognitive (baseline) testing, then a total of 22 hours of teaching and training given 1.5 hours twice a week for 2.5 to 3 months. Participants are provided with about four weeks of dynamic scanning training using the Neuro Vision Technology scanning device and about four weeks of mobility training using Neuro Vision Technology.

Dynamic scanning is investigated using the special light panel with 24 light bulbs divided into two rows, the panel length is corresponding to the 180 degrees that normal and well-functioning human eyes can see. When considered relevant, a participant is referred to an ophthalmologist, specialist optician, or a reading/visual clinic to ensure the optimal starting point for using the participant’s vision. Records are obtained and used to store test results.

Initially the Neuro Vision Technology assessment process uses presentations of a single light to determine a participant’s ability to perform spontaneous scanning of the affected field, and the participant’s degree of visual field loss. Then the participant will perform exercises with different complexities using sequences of multiple lights. A participant’s response to an exercise indicates his or her ability to attend to multiple stimuli, to systematically search the visual fields and how fast each participant is able to detect changes in the visual field. The Neuro Vision Technology is used to teach each participant visual search strategies from the perimeter of the affected visual field towards the intact visual field.

**Eligibility**

Inclusion criteria

- 14 years or older with brain injury
- persons with significant vision impairment
- eye sight must be 6/18 or better
- the time from symptoms onset to study inclusion is between 12 weeks and 3 years

Exclusion criteria

- severe cognitive dysfunction
- persons with anosognosia or severe neglect
  inability to move independently at least 35 meters with or without assistance, including wheelchairs
- inability to understand Danish or with communication disorders that prevent participation in tests
- terminal disorder, other progressive disorder
- significant abuse of alcohol or euphoric or narcotic drugs
- serious disorders such as mental illness, especially severe depression
  new brain injury or other significant disorders emerging after study inclusion
- impaired vision not due to brain damage, where the disorder is not considered to be permanent or where the field of vision does not cause significant disability

**Figure 2:**
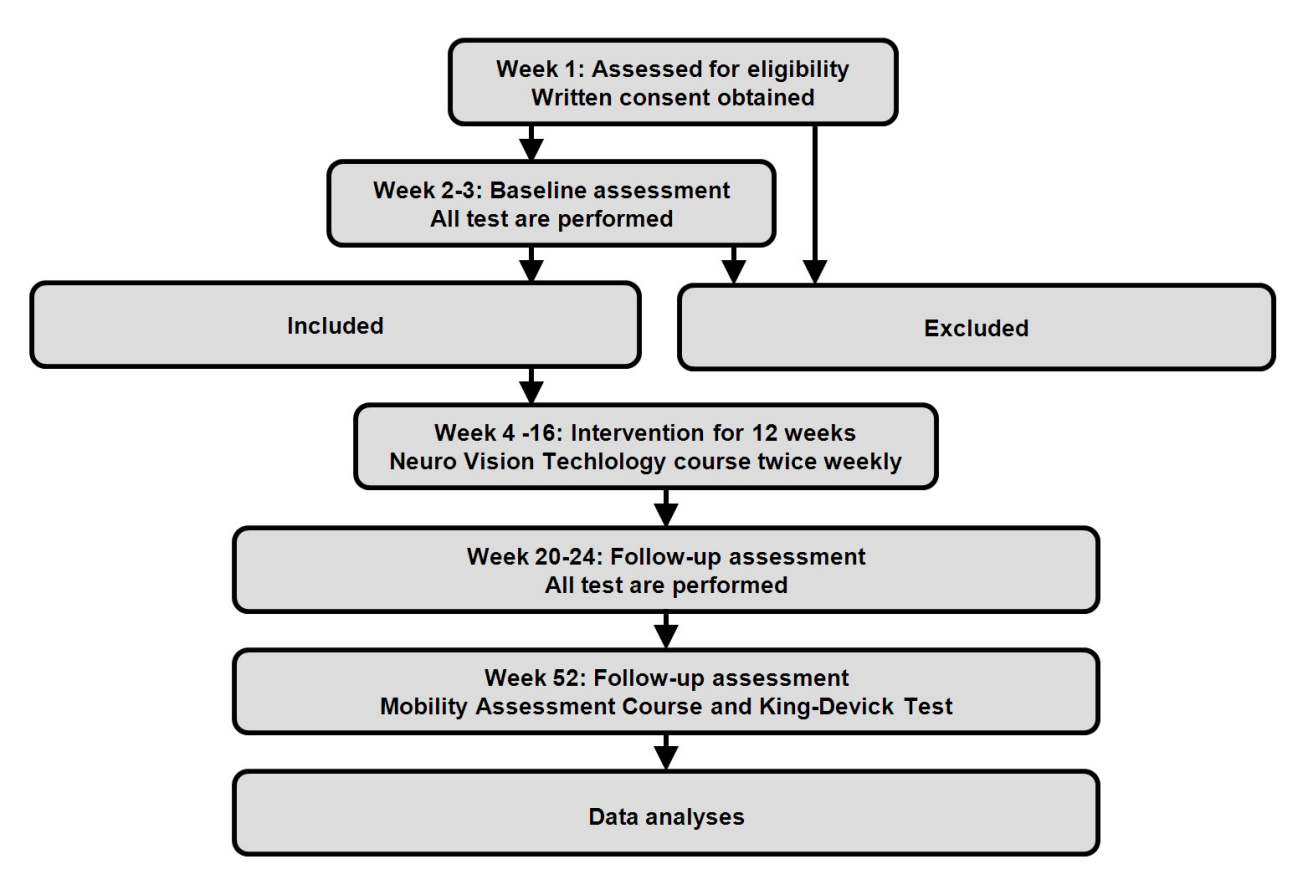
Flow Chart.

The Neuro Vision Technology training program is then used to transfer visual scanning strategies into the real world while doing therapist-assisted walking, with the aim of making participant perform these strategies automatically. Thus, scanning strategies are gradually implemented, learned and improved by participant first being exposed to quiet and then more busy locations, like moving participants from small and quiet rooms at our training facility into for example shopping centers or to a busy road, where participants have to safely cross at traffic light junctions safeguarded by a therapist. Depending on individual needs training may also involve the use of public transport, safely riding a bicycle and exercises in order to safely move in the traffic. A re-test of 3 hours duration is performed 6 weeks after the Neuro Vision Technology course has been finished and 52 weeks after a participant’s inclusion in the study (Figure 2).

All participants receive Neuro Vision Technology course training and are tested at baseline and at the end of the study for primary and secondary endpoints; the time frame is expected to be 5 to 6 months for study participation for each participant, although the Neuro Vision Technology course is ended after 4 months (Figure 2).

### Primary Hypothesis and Endpoint

It is our main hypothesis that participants will increase awareness of their surroundings, will improve navigating their surroundings, and will be able to experience a greater quality of life – including reduced symptoms of both fatigue and depression.

At the start of the intervention, standardized route training (mobility route training) is performed that participants must be able to perform independently, this intervention is also known as Mobility Assessment Course [2,6]. Mobility Assessment Course performance is the primary endpoint inspired by a similar trial, where the protocol was published, but currently the trial seems to have been cancelled [2]. On the route there are a number of targets (stars) that participants should locate. Investigators measure the number of targets ignored by any participant. Ability to perform correct target identifications by comparing how many targets participants identify before and after the intervention is the primary outcome measurement.

### Secondary endpoints

Time consumption in seconds and thus speed to complete the mobility route will be measured as a secondary endpoint. Investigators measure the time spent by participants to complete the route, and faster speeds indicate improved outcome as long as correct target identification has not been reduced.

Several other secondary endpoints will be used in the study. Visual functions will be estimated using the following five tests:

1. The National Eye Institute Visual Functioning Questionnaire-25 (VFQ-25), which is a questionnaire for measuring vision problems and concerns associated with a person’s visual function [7,8]. It has been shown that the VFQ-25 is a reliable and valid 25-item version of the 51-item National Eye Institute Visual Function Questionnaire (NEI-VFQ). The VFQ is useful in clinical trials where interview length is a consideration [9]. The VFQ-25 consists of 25 questions related to a person’s activities in daily life.
2. Test of Attentional Performance (TAP) Test 2.3 Visual Field will be used to test for visual field defects. During the test a participant must respond quickly to stimuli in a circumscribed area of the visual field (eg, whenever a flicker stimulus is presented at varying intervals). This happens while participants are engaged in a simultaneous central task to ensure that participants fixate on the middle of the screen throughout the entire test run. When the peripheral stimulus appears, the patient should press the reaction key as quickly as possible [10].
3. Behavioral Inattention Test will be used to assess attention and vision among participants [11]. Also known as Rivermead Behavioral Inattention Test, it is a battery of short tests to assess the presence and the extent of visual neglect on everyday problems faced by persons with visual inattention.  
4. Rey-Osterrieth’s complex figure test will be used but limited to copying an advanced visual figure (without recall) [12]. Rey-Osterrieth’s complex figure test is a well-known neuropsychological assessment in which participants are asked to reproduce a complicated drawing by copying it freehand. Many different cognitive abilities are needed for a correct performance, and the test therefore permits the evaluation of different functions, for example visuo-spatial abilities, attention, planning, and executive functions [12]. 
5. The King-Devick Saccade Test will be used as to estimate eye movement disorders, especially a participant’s ability to perform saccades and horizontal eye movements [13].

Cognitive abilities will be measured by the Montreal Cognitive Assessment (MoCA), which is a cognitive screening test providing an estimate of the intellectual functional level [14]. Originally the MoCA was designed as a short screening instrument for mild cognitive dysfunction and dementia. MoCA assesses cognitive domains, for example: attention, executive functions, working and long-term memory, language and visuo-constructional skills [14]. A score of 26 to 30 is considered normal, while lower scores may indicate cognitive dysfunction.

Participants will be screened for potential psychiatric and psychological disturbances as depression and debilitating fatigue using the following 3 questionnaires:

1. The Major Depression Inventory will be used to measure and estimate symptoms of depression, which have been present for the last 2 weeks. It is a 12-item self-report mood questionnaire, which was developed by the World Health Organization’s Collaborating Center in Mental Health [15].
2. The Fatigue Severity Scale is a 9-item self-administered questionnaire originally developed for measurements of fatigue in multiple sclerosis and systemic lupus erythomatosus. Each item is scored from 1 to 7 with higher scores reflecting increased levels of fatigue. In a recent study, investigators found more valid results if items 1 and 2 were excluded [16]. We refer to the reduced form as FSS-7 and will use the FSS-7 instead of the FSS to estimate fatigue in participants.
3. The Multidimensional Fatigue Inventory 20 (MFI-20), which is a 20-item self-administered questionnaire designed to measure 5 fatigue domains: general fatigue, physical fatigue, reduced activity, reduced motivation, and mental fatigue, will be performed. The MFI-20 general fatigue domain can be used as an overall measure of fatigue, and a score of 12 or more may indicate presence of debilitating fatigue [17-19].

Quality of life will be estimated using the European Quality of Life–5 Dimensions questionnaire (EuroQol-5D) with the health status from each subscale of the EuroQol-5D transformed into a single value by the use of population-based preference weights for Denmark [20]. The EuroQol-5D is applicable to a wide range of health conditions and treatments and provides a simple descriptive profile and a single index value for health status in participants. EuroQol-5D is designed for self-completion by participants and is ideally suited for use in clinics, and in face-to-face interviews [20].

For assessment of invalidity and ability to perform activities of daily living, the modified Barthel-100 Activities of Daily Living Index score will be used with scores ranging from 0 to 100. A score of 100 indicates no problems in general daily living activities, such as mobility, eating, personal hygiene, dressing and toilet use [21].

Furthermore, as part of the study, it will be assessed whether persons with lesion of right hemisphere (ie, left-field hemianopia) may be more likely to be disorientated and due to neglect will overlook more details - this is done by comparing trial participants with right vs. left hemispheric lesions.

### Blinding

Participants are not randomized as there is only an intervention group and no controls. As far as possible, it is ensured that the investigator who measures the subjects' functional level at the end of intervention has not previously had contact with, or knowledge of, the particular participant. Thus, the level of function of participants is evaluated by different persons before and after intervention to ensure impartial assessments.

### Sample Size Estimation

The primary endpoint is the ability to identify the highest number of correct targets on the mobility route test. Based on unpublished pilot data, we expect participants to correctly identify 40% of the mobility targets at baseline, and to be able to identify 65% of correct target 6 weeks after ending the Neuro Vision Technology course. As a basis for power calculation, alpha = .05 and beta = .2 are used. Sample size is calculated to 23 participants. Due to age, difficulty in transport, and the time-consuming intervention, up to 25% dropouts are expected. Thus, we aim to include 29 participants with permanent visual impairment after brain damage corresponding to hemianopia, quadrant hemianopia or scotoma resulting in functional impairment at activity or participation level.

### Data Collection

Data collection and databases are prepared for registration and data processing. Data at baseline include: age, living single vs cohabitant at home, in-home care services, mobility aids, social network, height, weight, smoking, and alcohol consumption.

### Statistical Analysis

Data comparisons of ranks before and after performances are calculated using Wilcoxon non-parametric test and optionally categorical variables with chi-2 test. Nonlinear correlations will be evaluated using the Spearman Rank correlation coefficient. Unused or incomplete data is not included in the statistical evaluation and is treated as missing data (ie, no calculations are made using such data). Lack of data is acceptable as long as an assessment of the primary endpoint is still possible. Data from participants that make it possible to assess the primary endpoint will be used as a minimum; however, we hope that data from all subjects can be used to evaluate secondary effect targets. *P* values of less than .05 will be considered significant.

### Ethics

The project is in accordance with the Helsinki Declaration, has been assessed by the Scientific Ethics Committee (protocol number H-17001534) and can be initiated. The ClinicalTrials.gov identifier is NCT03160131. The law of protecting personal data will be upheld.

There are no invasive treatments, and no adverse events or risks are expected in the trial. Regardless of the reason, any subject may at any time cease to participate in the trial.

## Results

Funding was provided in June 2017. Results are expected to be available in 2020. Sample size is calculated to 23 participants. Due to age, difficulty in transport, and the time-consuming intervention, up to 25% dropouts are expected; thus, we aim to include at least 29 participants.

## Discussion

### Overview

To the best of the authors’ knowledge, this is the first time an investigation will evaluate the effects of Neuro Vision Technology complementary vision rehabilitation and we have received all necessary funding. Previously a similar trial was announced, but there have been no updates on progress for years and we assume that trial may never be finished [2]. According to the Australia and New Zealand Clinical Trials Register no updates have been received regarding the before mentioned trial since June 16 2010. Furthermore, our broad spectrum of secondary endpoints ensures a thorough examination of possible changes in quality of life, beyond previous investigations and case studies [3,5].

### Limitations

Before planning the current trial, for years we evaluated the number of eligible participants, and finding eligible patients have been difficult. Therefore, we have omitted using a control group, since this may double the number of needed participants.

### Conclusion

This trial will evaluate the effects of Neuro Vision Technology rehabilitation therapy and provide answers to the efficacy of such treatment methods. Vision is the most important sense in humans; thus, even small permanent deficits can dramatically affect the quality of life and we hope to be able to improve compensatory treatments by our current investigation.

## Abbreviations

EuroQol-5D: European Quality of Life–5 Dimensions questionnaire
MCA: Montreal Cognitive Assessment
MFI-20: Multidimensional Fatigue Inventory 20
VFQ: Visual Functioning Questionnaire
